# Limited Evidence for Parent-of-Origin Effects in Inflammatory Bowel Disease Associated Loci

**DOI:** 10.1371/journal.pone.0045287

**Published:** 2012-09-27

**Authors:** Karin Fransen, Mitja Mitrovic, Cleo C. van Diemen, Thelma B.K., Ajit Sood, Andre Franke, Stefan Schreiber, Vandana Midha, Garima Juyal, Uros Potocnik, Jingyuan Fu, Ilja Nolte, Rinse K. Weersma

**Affiliations:** 1 Department of Genetics, University of Groningen, University Medical Centre Groningen, Groningen, The Netherlands; 2 Department of Gastroenterology and Hepatology, University of Groningen, University Medical Centre Groningen, Groningen, The Netherlands; 3 Center for Human Molecular Genetics and Pharmacogenomics, Medical Faculty, University of Maribor, Maribor, Slovenia; 4 Department of Genetics, University of Delhi, South Campus, New Delhi, India; 5 Department of Medicine, Dayanand Medical College and Hospital, Ludhiana, India; 6 Institute for Clinical Molecular Biology, Christian-Albrechts-University, Kiel, Germany; 7 Department of General Internal Medicine, University Clinic Schleswig-Holstein, Kiel, Germany; 8 Department of Epidemiology, University of Groningen, University Medical Centre Groningen, Groningen, The Netherlands; 9 Faculty of Chemistry and Chemical Engineering, University of Maribor, Maribor, Slovenia; University of California, Irvine, United States of America

## Abstract

**Background:**

Genome-wide association studies of two main forms of inflammatory bowel diseases (IBD), Crohn’s disease (CD) and ulcerative colitis (UC), have identified 99 susceptibility loci, but these explain only ∼23% of the genetic risk. Part of the ‘hidden heritability’ could be in transmissible genetic effects in which mRNA expression in the offspring depends on the parental origin of the allele (genomic imprinting), since children whose mothers have CD are more often affected than children with affected fathers. We analyzed parent-of-origin (POO) effects in Dutch and Indian cohorts of IBD patients.

**Methods:**

We selected 28 genetic loci associated with both CD and UC, and tested them for POO effects in 181 Dutch IBD case-parent trios. Three susceptibility variants in *NOD2* were tested in 111 CD trios and a significant finding was re-evaluated in 598 German trios. The UC-associated gene, *BTNL2*, reportedly imprinted, was tested in 70 Dutch UC trios. Finally, we used 62 independent Indian UC trios to test POO effects of five established Indian UC risk loci.

**Results:**

We identified POO effects for *NOD2* (L1007fs; OR = 21.0, P-value = 0.013) for CD; these results could not be replicated in an independent cohort (OR = 0.97, P-value = 0.95). A POO effect in IBD was observed for *IL12B* (OR = 3.2, P-value = 0.019) and *PRDM1* (OR = 5.6, P-value = 0.04). In the Indian trios the *IL10* locus showed a POO effect (OR = 0.2, P-value = 0.03).

**Conclusions:**

Little is known about the effect of genomic imprinting in complex diseases such as IBD. We present limited evidence for POO effects for the tested IBD loci. POO effects explain part of the hidden heritability for complex genetic diseases but need to be investigated further.

## Introduction

Crohn’s disease (CD) and ulcerative colitis (UC) are the two main forms of chronic relapsing inflammatory bowel diseases (IBD). With a cumulative prevalence of up to 800 per 100,000 in Europe and 570 in North America [1], it is considered one of the most common immune-related diseases worldwide. Typically, from their second or third decade on, patients suffer from a chronic relapsing inflammation of the gut, which is often accompanied by extra-intestinal manifestations and complications that can be extremely debilitating and severe. Treatments are costly and often insufficient and can be accompanied by severe side-effects [2]. Hence, there is an urgent need for new therapeutic targets and curative medication. The pathogenesis is largely unknown and it is currently thought that an aberrant immune response to commensal microflora in a genetically susceptible host underlies the disease [3].

Prior to the introduction of genome-wide association studies (GWAS), only three loci had been consistently associated with either form of IBD. Over the past six years, multiple GWAS and meta-analyses have yielded a lengthening list of variants associated with CD (71 confirmed independent genetic risk loci) and UC (47 loci) [4], [5]. Nevertheless, despite this encouraging progress, as much as 77% of the estimated heritability for CD and 72% for UC is still considered to be unexplained [4], [5]. Thus, one of the challenges in the post-GWAS era is to identify potential sources of this ‘hidden heritability’ [6], which may reside in associated variants with lower odds ratios, gene-gene interactions, gene-environment interactions, and/or structural variation.

**Figure 1:**
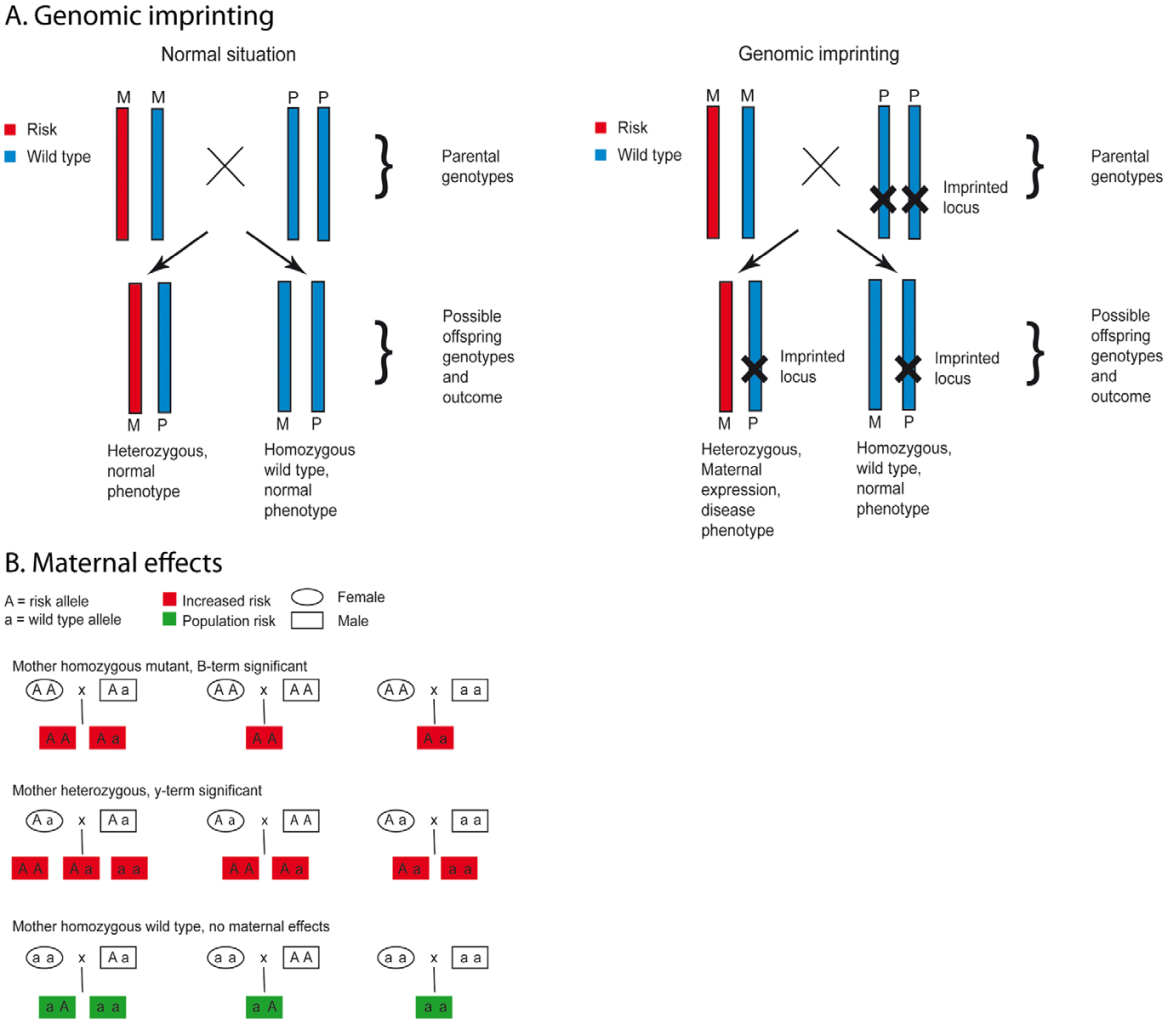
Distinct types of parent-of-origin mechanisms tested in this study. Fig. 1a. Genomic imprinting: Genomic imprinting is characterized by consequent silencing of one allele, depending on the parental origin. In the example shown above a normal situation is displayed on the left and the genomic imprinting is shown on the right; red is the risk allele and bleu is the wild type allele. The maternal genotype is heterozygous, the father’s genotype is homozygous wild-type. Offspring in the left scenario have a normal phenotype since the paternal wild-type allele is expressed in the heterozygous offspring and the mutated allele of the mother is thus rescued by the paternal allele. On the right genomic imprinting is shown, reflecting the α-term in the method used to test for parent of origin effects. In this example there is a significant genomic imprinting effect and the OR >1 so the paternal allele is silenced (see materials and methods section statistical analysis). We assume an additive or recessive model of inheritance. Two possible outcomes are listed, if the offspring inherits the risk allele from the mother and the wild-type allele from the father is subjected to genomic imprinting, then only the risk allele is expressed, thus the offspring is affected by the mutated allele from the mother. Fig. 1b. Maternal effects: Maternal effects are effects of the maternal genotype on the fetal phenotype, irrespective of the fetal genotype, these effects are reflected by the β- and γ-terms in the likelihood ratio test that was used to test for parent of origin effects in our study. In the example given above, the β- and γ-terms are significant with an OR >1, meaning that the risk of disease is higher if the mother carries two or one risk allele respectively. A recessive or co-dominant model is assumed, and higher expression of the mutant allele leads to disease. If the genotype of the offspring is red, then maternal effects cause increased disease risk and if it is green than the normal population risk applies. If the mother is homozygous wild-type, no maternal effects occur. If she is homozygous mutant or heterozygous for the risk allele, the offspring is subjected to maternal effects and thus has an increased disease risk. Note that the wild-type homozygous offspring has a higher disease risk if both parents are heterozygous.

In addition, parent-of-origin effects (POO) may comprise a piece of the missing heritability puzzle in IBD, as suggested by Akolkar et al. [7]. They show that offspring of mothers with CD are at higher risk for CD than when fathers are affected. More recently, Zelinkova et al. showed there was maternal imprinting and female predominance in familial Crohn’s disease [8]. This could be explained with at least two distinct types of POO mechanisms (Fig. 1). If the paternal allele is inactivated by genomic imprinting, then expression of the locus is determined only by the maternal allele (Fig. 1a). If this effect is not taken into account, there may be a significant loss in the statistical power of genetic association studies [9], [10]. Secondly, maternal effects such as diet or genotype affect the environment for the developing fetus (Fig. 1b). It is thought that maternal proteins or circulating RNA passes the placental barrier and may cause changes in the epigenome of fetal DNA, thereby influence its phenotype.

In this study, we tested for these two types of POO effects in IBD by using a likelihood ratio test developed by Weinberg [11]. A previous version of this method (parental asymmetry test) has already successfully identified a POO effect in another complex genetic disease, type 1 diabetes [12].

## Materials and Methods

### Ethical Considerations

This study was approved by the institutional review boards (Institutional ethical committee, Dayanand Medical College and Hospital, Ludhiana and Institutional ethical committee, University of Delhi South Campus; Ethical Review Board of the Medical Faculty of the Christian-Albrechts-University of Kiel; Institutional review board, University Medical Centre Groningen, The Netherlands) of each of the hospitals and written informed consent was obtained from all subjects personally.

### Subjects

All patients were diagnosed according to standard clinical criteria by endoscopy, radiology and histopathology [13]. A total of 249 classical offspring-parent trios (one affected offspring with two unaffected parents) were included in our initial POO analysis. Of these trios, 115 offspring had CD and 134 had UC. All 115 CD trios were of western European descent from the Netherlands and were collected at the IBD Center in the Department of Gastroenterology and Hepatology, University Medical Center Groningen (UMCG), the Netherlands. For UC there were 72 trios of Dutch ancestry (collected at the UMCG) and 62 of Indian ancestry (collected at Dayanand Medical College and Hospital, Ludhiana, Punjab, India). Tables 1 and 2 show the clinical characteristics of the studied cohorts. Genotype data of an independent replication cohort, consisting of 598 CD parent-offspring trios from Germany, were obtained for the *NOD2* L1007fs variant from the Institute of Clinical Molecular Biology, Christian-Albrechts-University of Kiel, Germany. These data were available from a previous study, no phenotypic data is available [14].

**Table 1:** Phenotypic characterization of subjects with Crohn’s disease.

Cohort	Number of trios	No. of males (%)	AOO	Ileal	Colonic	Ileocolon	Upper GI
Dutch CD	115	39 (34%)	24	23	28	64	11
German CD	598	N/A	N/A	N/A	N/A	N/A	N/A

**Table 2:** Phenotypic characterization of subjects with ulcerative colitis.

Cohort	Number of trios	No. of males (%)	AOO	Proctitis	Left-sided	Extended	Unknown
Dutch UC	72	30 (42%)	25	8	19	38	5
Indian UC	62	45 (73%)	28	22	15	20	5

N/A not available, AOO average age of onset. Cases and disease location are given according to the Montreal classification. for CD L1, L2, L3 and L4; for UC E1, E2, E3. No phenotypic information was available for the German cohort.

### Genotyping and Quality Control

All Dutch and Indian subjects were genotyped using the Illumina Immunochip (iCHIP) (Illumina Inc., San Diego, California, United States of America), which is a custom-made genotyping array that contains ∼200,000 single nucleotide polymorphisms (SNPs) focusing on immune-mediated diseases [15], [16]. Genotyping was performed according to the manufacturer’s protocol. Genotyping clusters of the SNPs included in the current analysis were checked manually using GenomeStudio software by Illumina Inc. [15], [16]. Individuals with a call rate <95% and/or discordant gender information, and SNPs with a call rate <98% were removed from further analysis. Identity-by-descent analysis by Plink software was used to test for incorrect family relations (Mendelian errors) in the trios, but no mismatches were identified.

### SNP Selection

For this study we used several strategies to select SNPs. First, to gain power we pooled the Dutch UC and CD trios and tested for POO effects in 28 established IBD loci that are both associated to CD and UC [4]. To avoid losing significance due to multiple testing correction we tested the 28 overlapping loci instead of all 99 associated risk loci. Variant rs736289 is not present on iCHIP and no proxy (r^2^>0.5) could be found. Two variants (rs12261843, rs181359) were also not captured by iCHIP, but we identified perfect proxies using SNAP software: rs12261843 was represented by rs12254167 (r^2^ = 1; D’ = 1) and rs181359 was represented by rs2266961 (r^2^ = 1; D’ = 1) [17]. Second, we aimed to test for the existence of POO effects in the UC risk SNPs established in Indians [18]. In addition, we aimed to include SNPs from known imprinted genes in our analysis. For this, a publicly available database of known imprinted genes was compared with all 99 IBD-associated loci [19]. The associated locus was defined as the region of r^2^>0.5 flanking the most significantly associated SNP, then extended to the nearest recombination hot-spot, and from there for an extra 100 kb. Comparison of IBD-associated genes with the known imprinted genes resulted in the inclusion of one extra gene, *BTNL2*; since this is a UC-specific locus it was only analyzed in the Dutch and Indian UC cohorts. SNP rs9268853 is the reported UC risk SNP in the Caucasians and was tested in the Dutch trios, rs3763313 was tested for POO effect in the Indian trios since this is the reported risk SNP in the Indian population. Lastly, we included *NOD2* since it is the most strongly associated gene and is most replicated in association studies of CD in populations of western European descent. Three common disease-susceptibility variants (G809R, R702W, and L1007fs) were therefore tested for POO effects in the Dutch CD trios, and subsequently the L1007fs variant was tested in the German replication cohort [20].

### Statistical Analysis

A power analysis was performed with Quanto software [21] and showed that in Dutch trios (n = 181) we had more than 80% power to detect POO effects of OR ≥3 in variants with MAF ≥0.025. In Indian trios (n = 62) we had 80% power to detect POO effects of OR ≥3 in variants with MAF ≥0.075 (see Fig. S1). POO effects were calculated by a log-likelihood ratio test, which is a statistical test used to compare the fit of the null hypothesis (i.e. no evidence/presence of POO in our case) and the alternative hypothesis. The test is based on the likelihood ratio, which expresses how many times more likely the data are under one model than the other and can be used to decide whether to reject the null model in favor of the alternative model. Weinberg *et al.* [11] have developed a log-linear model when a case-parents triad is genotyped and jointly classified according to the number of copies of a particular allele carried by the mother, father, and child (denoted as “M,” “P,” and “C,” respectively), there are 15 possible outcomes (i.e. mating types). The family-specific outcomes (i.e., the cell into which a particular triad is classified) are independent, provided that each family contributes only one case. The counts based on classification of the triads studied can therefore be thought of as distributed according to a 15-cell multinomial. The method is based on consideration of mating types in which the mother and father carry unequally many copies of the variant allele, with further stratification on the number of inherited copies of the allele, C. This second level of conditioning (on C) effectively removes any effects related jointly to the inherited number of copies and the parental-allele counts M, P. In short, the method is valid if inheritance of allele is Mendelian, if there is parental symmetry within mating types in the population studied, and if the gene under study is not in linkage disequilibrium with another disease-susceptibility gene. First, the relative penetration of the risk allele of the child is established by determining the parental origin of the risk allele. In the latter, the difference in disease risk is compared for the varying amounts of risk alleles carried by the mothers; the genotype of the child is not relevant. The likelihood ratio test calculates α-, ß-, and γ-terms. The α-term indicates the significance level for genomic imprinting effects: if OR >1, the risk allele is transmitted more often from the mother to the patient and if OR <1 then it is transmitted more often from the father. The ί- and γ-terms indicate the prenatal effect of the maternal genotype when the mother carries two risk alleles or one risk allele, respectively. When the ß- or γ-term is significant and OR >1 then the child has more chance of getting the disease due to maternal effects. If OR <1 then the child has less chance of developing the disease as a consequence of this prenatal effect. Bonferroni multiple testing corrections were applied to the four different analyses.

## Results

DNA of 249 complete IBD trios was available for our study, of which four CD (4/115) and two Dutch UC trios (2/72) did not pass the quality control. Therefore 243 IBD trios (111 CD, 70 Dutch UC & 62 Indian UC) were available for the discovery phase of the study. Our findings were then replicated in an independent replication cohort consisting of 598 German CD trios.

### Parent-of-origin Analysis in Dutch IBD Trios

A nominally significant genomic imprinting effect was found in the *IL12B* gene (α term: P = 0.019; OR = 3.2), with OR >1 indicating that the risk allele is more often transmitted from the mother to the child. In addition, the β term was nominally significant (p-value = 0.003; OR = 0.2) with OR <1, indicating that offspring have less chance of getting the disease if their mothers carry two risk alleles. The *PRDM1* gene showed a nominally significant maternal effect if the mother carried two risk alleles (β term: p-value = 0.04; OR = 5.6), with OR >1 indicating that the offspring have more chance of getting the disease. The other tests did not result in significant POO effects. However, none of these associations were significant after the Bonferroni correction (table 3).

**Table 3:** Results of the parent-of-origin (POO) analysis of Dutch IBD Trios (n = 181) for the 28 known SNPs shared between ulcerative colitis and Crohn’s disease.

SNP	Gene		RA		p-α		OR-α		p-ß		OR-ß		p-γ		OR-γ			
rs11209026		*IL23R*		G		0.6		0.6		1.0		1.6		1.0		1.0		
rs7554511		*KIF21B*		C		0.2		1.7		0.5		0.7		0.6		0.8		
rs3024505		*IL10*		A		0.4		1.5		0.4		0.5		0.3		1.5		
rs7608910		*REL*		G		0.4		0.7		0.7		0.8		0.8		0.9		
rs2310173		*IL1R2*		T		0.5		1.4		0.3		0.5		0.7		0.9		
rs3197999		*MST1*		A		0.5		0.8		0.6		1.4		0.3		1.4		
rs6451493	*PTGER4*		T		0.1		2		0.5		1.6		0.4		1.6			
**rs6871626**	***IL12B^§^***		A		**0.019**		**3.2**		**0.003**		**0.2**		0.2		0.6			
rs6556412		*IL12B* ***^§^***		A		1.0		1.0		0.9		1.0		0.9		0.9		
rs6908425	*CDKAL1*		C		0.5		0.7		0.8		0.8		0.6		0.7			
**rs6911490**	***PRDM1***		T		0.2		0.5		**0.04**		**5.6**		0.6		1.2			
rs10758669		*JAK2*		C		1.0		1.0		0.2		0.5		0.8		0.8		
rs4246905		*TNFSF15*		C		0.8		0.9		0.8		0.8		0.8		0.9		
rs10781499		*CARD9*		A		0.8		1.0		0.5		0.7		0.4		0.8		
rs12254167		CREM CCNY*		N/A		0.1		0.5		0.6		1.4		0.3		1.4		
rs10761659		*ZNF365*		G		0.4		1.4		0.8		0.9		0.9		0.9		
rs6584283		*NKX2-3*		T		0.3		0.6		0.4		1.6		0.1		1.9		
rs2155219		*C11orf30*		T		0.1		0.5		0.3		1.8		0.6		1.2		
rs17293632		*SMAD3*		T		0.2		1.8		0.1		0.4		0.3		0.7		
rs2872507		*ORMDL3*		A		0.8		0.9		0.3		1.7		0.6		1.2		
rs1893217		*PTPN2*		G		0.1		2.1		0.7		1.2		0.3		0.7		
rs12720356		*TYK2*		C		0.6		1.4		0.5		0.5		0.9		0.9		
rs2297441		*RTEL1-SLC2A4RG*		A		0.7		0.8		0.9		1.1		0.9		0.9		
rs1297265		*intergenic*		A		0.9		1.1		0.3		1.7		0.3		1.5		
rs2836878		*intergenic*		G		0.6		1.3		0.9		0.9		0.6		1.3		
rs2838519		*ICOSLG*		G		0.7		0.8		0.3		1.7		0.8		0.9		
rs2266961		YDJC*		N/A		0.9		1.0		0.8		0.8		0.5		0.8		

P-value (p-α; ß; γ) and odds ratio (OR-α; ß; γ) of the alpha-, beta-, and gamma-terms. Alpha-term indicates the genomic imprinting effect; Beta-term and gamma-term indicate the maternal effect in case the mother carries respectively two and one risk alleles. N/A not available. Significant associations are shown in bold. P-values displayed in the table are not corrected for multiple testing. *reported SNP not present/captured by the Immunochip, a proxy was used, therefore no risk allele could be reported. r^2^ = 1; ***^§^***r^2^ = 0.03; two independent hits in one gene.

### NOD2 in Dutch and German CD Trios

Three established CD variants in *NOD2* (G809R, R702W, L1007fs) were tested for POO effect in the 111 CD trios [20]. After correcting for multiple testing, a significant genomic imprinting effect was detected for the L1007fs variant (α term: p-value = 0.013; OR = 21.0). The risk allele was transmitted more often from the mother than the father. Given the high OR we aimed to replicate this finding in an independent German cohort for which *NOD2* genotyping data was available. Unfortunately, our results could not be replicated in this cohort (α term: p-value = 0.95; OR = 0.97) (table 4).

**Table 4:** Results of the parent-of-origin (POO) analysis for the NOD2 variants in Dutch Crohn’s disease trios (n = 111) and replication in German Crohn’s disease trios (n = 598).

SNP	Gene		p-α		p-α replication		OR-α		p-ß		p-ß replication		OR-ß		p-γ		p-γ replication		OR-γ		
G908R		*NOD2*		0.1				9.0		N/A*				N/A*		0.2				0.3	
R702W	*NOD2*		0.4				2.3		1.0				0		0.2				0.4		
**L1007fs**	***NOD2***		**0.01** ◊		0.9		**21.0**		1.0		1.0		7.7		0.1		0.8		0.2		

P-value (p-α; ß; γ) and odds ratio (OR-α; ß; γ) of the alpha-, beta-, and gamma-terms. P-value of the replication study (p- α; -ß; - γ replication ) of the alpha-, beta-, and gamma-terms**.** Alpha-term indicates the genomic imprinting effect; Beta-term and gamma-term indicate the maternal effect in case the mother carries respectively two and one risk alleles. Significant associations are in bold.

◊ Significant after Bonferroni multiple testing correction. P-values displayed in the table are not corrected for multiple testing.

* No homozygous mothers are available for beta-term analysis.

### Known Imprinted Gene BTNL2 in Dutch UC Trios

No significant POO effects were detected the established UC SNP in the *BTNL2* locus (rs9268853) in 72 Dutch UC trios (table 5).

**Table 5:** Results of the parent-of-origin (POO) analysis in the BTNL2 locus in Dutch UC ulcerative colitis trios (n = 72).

SNP		Gene	RA		p-α		OR-α		p-ß		OR-ß		p-γ		OR-γ		
rs9268853	*BTNL2*			T		1.0		1.0		0.7		0.7		0.6		0.6	

P-value (p-α; ß; γ) and odds ratio (OR-α; ß; γ) of the alpha-, beta-, and gamma-term. Alpha-term indicates the genomic imprinting effect; Beta-term and gamma-term indicate the maternal effect in case the mother carries respectively two and one risk alleles. Significant associations are in bold. P-values displayed in the table are not corrected for multiple testing.

### Indian UC Analysis

The established Indian UC SNPs were tested for POO effects in 62 Indian trios [18]. We found a nominally significant genomic imprinting effect in the *IL10* locus (p-value = 0.03; OR = 0.16) where the OR <1 indicates that the risk allele is more often transmitted from the father. This association does not, however, pass the multiple testing correction. This SNP was also tested in the population of western European descent and we could not detect any significant imprinting effect. The *NOD2* variant that showed association in the Indian population could not be tested for POO effects since only homozygous wild-type fathers were available, hence all the trios were uninformative (table 6).

**Table 6:** Results of the parent-of-origin (POO) analysis in Indian UC trios (n = 62).

SNP	Gene	RA	p-α	OR-α	p-ß	OR-ß	p-γ	OR-γ
rs6426833	*RNF186*	A	0.2	0.3	0.2	4.3	0.1	4.8
**rs3024505**	***IL10***	A	**0.03**	**0.16**	0.8	1.3	0.5	1.5
rs3763313	*BTNL2*	T	0.8	0.8	1.0	2.0	1.0	4.0
rs2395185	*HLA-DRA*	A	0.3	2.3	0.8	1.3	0.4	3.0

P-value (p-α; ß; γ) and odds ratio (OR-α; ß; γ) of the alpha-, beta-, and gamma-term. Alpha-term indicates the genomic imprinting effect; Beta-term and gamma-term indicate the maternal effect in case the mother carries respectively two and one risk alleles. Significant associations are in bold. P-values displayed in the table are not corrected for multiple testing.

## Discussion

For the first time parent-of-origin effects have been tested in IBD on a genetic level for the overlapping IBD-associated loci. We found limited evidence that POO effects exist in IBD in the Dutch population for *IL12B*, *PRDM1* and *NOD2* in our discovery cohort, but the large POO effect for *NOD2* could not be replicated in an independent German replication cohort. Moreover, we found a nominally significant POO effect in *IL10* in our Indian population. Although the results from the Dutch trios might be false-positive, they imply that the paternal allele has been silenced and thus does not increase the disease risk in all genes for which we found a POO effect. This is consistent with results from epidemiological studies that show that IBD is transmitted to offspring more often from the mother than the father.

*NOD2* is the most strongly associated and most consistently replicated CD gene. Here we observed a genomic imprinting effect for the L1007fs mutation in Dutch CD trios, yet we failed to replicate this in an independent German cohort. The results in our initial analysis might be a false-positive finding, although we had sufficient power to detect effects in the Dutch trios. Although both cohorts were of western European descent, we question whether population specific and environmental factors might play a major role in POO effects and explain part of the lack of replication. We will further elaborate on this later in the discussion. TheL1007fs mutation seems to have a predominant role in CD families since recently it has been shown in a case report that all family members were carrying the mutation and had CD [22]. Moreover in cases of homozygosity this variant will lead to ileal stenosis [23], [24], implying a strong effect of the L1007fs mutation on the disease phenotype.

Our results suggest a possible contradictory effect of the two types of POO effects we studied for the *IL12B* gene both with a nominal significance. The genomic imprinting analysis (α-term) showed inheritance of the disease risk from the mother, while the analysis of independent maternal effect showed protection for disease if the mother carries two risk alleles (β-term). This suggests that the maternal risk allele is expressed and causes a higher disease risk, but simultaneously and independently, if the mother carries two risk alleles the child has a lower risk for IBD due to *in utero* effects on the fetus. *IL12B* resides on the established and consistently replicated IBD locus on chromosome 5q33 and it encodes a sub-unit of IL23, which is involved in Th17/IL23R signaling. This pathway has been implicated in several chronic, immune-related diseases such as psoriasis, rheumatoid arthritis, and ankylosing spondylitis [25], [26], [27].

The *PRDM1* gene showed a nominally significant maternal effect in the POO analysis in the population of western European descent when mothers carried two risk alleles; the OR of 5.6 supports results from others that IBD is more often transmitted from the mother than the father. The environment in which the fetus develops causes changes that increase the risk for CD. *PRDM1* has been associated to several immune-related diseases, but also to various types of lymphomas [28], [29], [30], [31]. It encodes a protein that represses the expression of the β-interferon gene. Hypothetically, if the maternal effect causes altered expression of *PRDM1,* an aberrant immune response could increase CD risk.

In the Indian trio study we found a nominally significant genomic imprinting effect for the *IL10* locus. In contrast to the findings in the population of western European descent, the risk allele was more often transmitted from the father to the child. No epidemiological studies of the Indian population are available to validate the paternal transmission. We could not replicate the POO effects from the Dutch population in the Indian trios nor *vice versa*. This might indicate that POO effects are population specific. Later in the discussion we will discuss this in further detail.

Weinberg’s method to detect POO effect is a robust one. Moreover it takes genomic imprinting and maternal effects into consideration simultaneously. It therefore has less power than the standard parental asymmetry test (PAT) that only tests for genomic imprinting, but PAT is invalid if maternal effects are present [11]. The importance of these maternal effects has been shown in mice, with a knockout of the serotonin 1A receptor gene leading to an anxiety-like phenotype. Implantation of wild-type embryos into knockout mothers and cross-fostering of the pups with wild-type mothers showed the full anxiety-phenotype, indicating that the maternal genotype influences the phenotype and that this effect persists after birth [32]. This is supported by our evidence for maternal effects in the *PRDM1* and *IL12B* loci.

We do not know why POO effects occur. Humans are diploid organisms and as such, can survive the, on average, 500 recessive mutations that are present in every human being, since most deleterious effects are rescued by the other allele [33]. Genomic imprinting significantly deduces diploidy by consequently inactivating one haplotype depending on its parental origin and thus impairing the rescue mechanism. The most cited and best supported hypothesis for the existence of this counter-intuitive phenomenon is the parental conflict hypothesis, in which both sexes have a need to pass on their genetic information to the next generation. Yet this does not explain the existence of genomic imprinting in immune-related genes, for example [34]. Hypothetically, to prevent adverse reactions passing from mother to fetus, it is important that the immune responses are alike and thus preferably maternal immune genes are expressed.

In our study we had sufficient power (>80%) to detect POO effects with an OR of three or higher for each SNP in our study. By adding more tests the significance level must be adjusted accordingly and the power to detect differences is lower. Therefore we chose to only test the 28 overlapping IBD risk loci in a pooled cohort of Dutch CD and UC trios instead of all 99 risk loci. Consequently, bigger cohorts are needed to test the remaining IBD loci for POO effects.

None of our findings in one population could be replicated in another population. At least three reasons could explain this fact. First, it might be that the initial findings are false positive findings: the cohorts have a limited size and thus more variation around the mean, resulting in a higher chance of false positive findings. Second, it is unknown for how long genomic imprinting effects are stable in humans. It has been shown from mouse studies that genomic imprinting is stable for at least 3 generations [35]. No data is available in human studies. Moreover, genomic imprinting was shown to be influenced by environmental factors [36], [37], which could mean that although the imprinting mechanism is global, distinct genes may be imprinted in different populations because they were exposed to distinct environmental effects. The latter two could indicate that even within populations different imprinting effects occur.

In conclusion, we aimed to identify genomic imprinting effects and maternal effects acting on the risk alleles of IBD and we showed, for the first time, that *IL12B*, *NOD2* and *PRDM1* might be involved in these phenomena in Dutch IBD trios. It has already been shown that POO effects exist in type 1- and type 2 diabetes, which like IBD are complex genetic disorders and show a substantial overlap of disease susceptibility loci with IBD [10], [12]. Given the high OR in *NOD2* we sought to replicate our findings, but could not confirm the POO effect for *NOD2* in an independent German replication cohort. In the Indian population we did identify POO effects in the *IL10* gene. We could neither replicate our findings from the Dutch trios in the Indian or in the German population nor our findings from the Indian population in the Dutch cohort. This suggests that POO effects are either false-positive findings or prone to be population specific. We anticipate that future investigations, using larger, multi-ethnical cohorts will help to shed light on these complex and currently little known relationships. Parent-of-origin effects can take various forms and are not restricted to imprinting, but may involve a variety of mechanisms including gender effects, epistasis, epigenetic effects, and environmental influences during pre- or postnatal development. Better understanding of such effects will probably require detailed studies of model organisms in which breeding and environment can be carefully controlled. Given that alleles identified through GWAS account for a relatively small fraction of heritability, parent of origin effects may underlie some of the missing heritability problem. With appropriate family-based study designs data analysis methods and international collaborative efforts it will be possible to screen for parent of origin effects across the entire genome. In addition, epigenetic profiling on a genome scale will likely lead to the identification of novel epigenetic marks in a variety of disorders that may provide a bridge among the parental genome, parental environment, and offspring phenotype. We anticipate that the investigations of alternative models of inheritance, appropriate study design and application of novel technologies will enable a more complete picture of heritability in human traits, leading to new insights in the field of genetics of complex diseases.

## Supporting Information

Figure S1 **Power analysis of a. the Dutch trio analysis (181 trios) and b. the Indian trios (62 trios).** S1a. Power calculation of the Dutch trio analysis: The power is shown on the x-axis, the different odds ratios (OR) are shown on the y-axis. The different lines represent SNPs with different minor allele frequencies. In red, the regular 80% power cut-off is shown. With an OR of 3, we have sufficient power to detect parent-of-origin effects in SNPs with a MAF of 2.5%. S1b. Power calculation of the Indian trio analysis: The power is shown on the x-axis, the different odds ratios (OR) are shown on the y-axis. The different lines represent SNPs with different minor allele frequencies. In red, the 80% power cut-off is shown. With an OR of 4, we have sufficient power to detect parent-of-origin effects in SNPs with a MAF of 4.0%.
(TIF)
