# Supplementary figures and images for: Limited Evidence for Parent-of-Origin Effects in Inflammatory Bowel Disease Associated Loci
Source: PLoS One. 2012 Sep 27;7(9):e45287. doi: 10.1371/journal.pone.0045287 (PMC3459955; doi:10.1371/journal.pone.0045287)

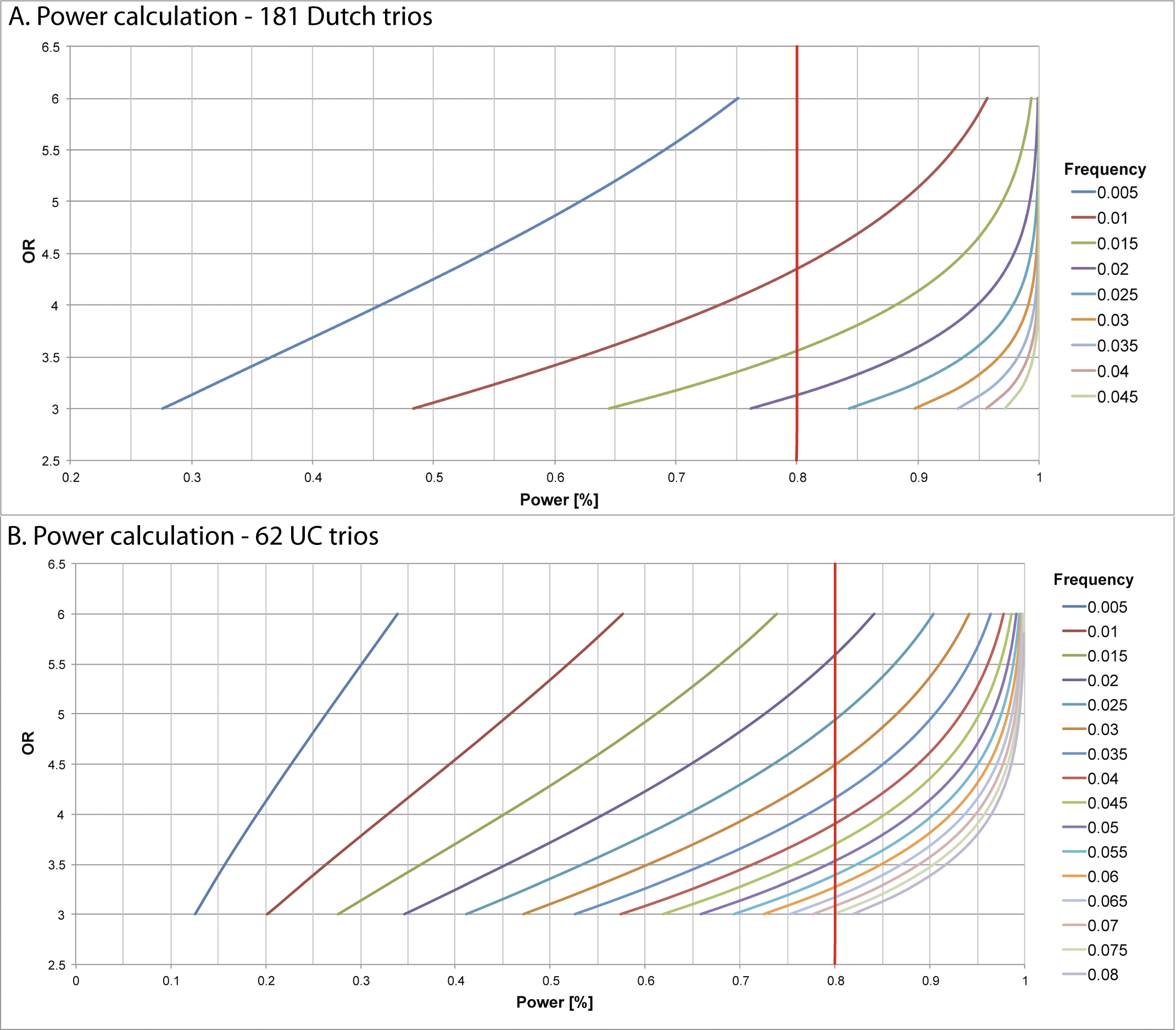

Supplement: Figure S1 — Power analysis of a. the Dutch trio analysis (181 trios) and b. the Indian trios (62 trios). S1a. Power calculation of the Dutch trio analysis: The power is shown on the x-axis, the different odds ratios (OR) are shown on the y-axis. The different lines represent SNPs with different minor allele frequencies. In red, the regular 80% power cut-off is shown. With an OR of 3, we have sufficient power to detect parent-of-origin effects in SNPs with a MAF of 2.5%. S1b. Power calculation of the Indian trio analysis: The power is shown on the x-axis, the different odds ratios (OR) are shown on the y-axis. The different lines represent SNPs with different minor allele frequencies. In red, the 80% power cut-off is shown. With an OR of 4, we have sufficient power to detect parent-of-origin effects in SNPs with a MAF of 4.0%. (TIF) [file pone.0045287.s001.tif]
